# Dr. Robert Nasmyth

**Published:** 1858-04

**Authors:** 


We learn with sincere sorrow, from the January number of the London Quarterly Journal of Dental Science, that Dr. ROBERT NASMYTH died recently at the residence of his father, in Edinburgh. A few years only have elapsed, since we were called upon to announce the death of his lamented uncle, Dr. Alexander Nasmyth, and two or three years later, we had the pleasure of making the acquaintance of his nephew, while on a visit to this country—and we little thought, at that time, we should be called upon so soon to record his decease. During the week or two which he spent in Baltimore, the senior editor, from the noble qualities both of heart and mind which he exhibited, became warmly attached to him, and most sincerely does he sympathize with his bereaved family. Dr. N. was a young man of high promise, and had his life been spared, he would soon have become a distinguished member of the profession. He had only been in practice a few years at the time of his decease.

